# The Latest Advances in Rosacea Treatment: A Systematic Review

**DOI:** 10.3390/ph19070982

**Published:** 2026-06-24

**Authors:** Anastazja Andrusiewicz, Sofiia Khimuk, Jakub Niżnik, Dmytro Sirko, Daniel Mijas, Danuta Nowicka

**Affiliations:** 1 Faculty of Medicine, Wroclaw Medical University, 50-367 Wroclaw, Polandsofiia.khimuk@student.umw.edu.pl (S.K.); jakub.niznik@student.umw.edu.pl (J.N.); dmytro.sirko@student.umw.edu.pl (D.S.); daniel.mijas@student.umw.edu.pl (D.M.); 2 University Centre of General Dermatology and Oncodermatology, Faculty of Medicine, Wroclaw Medical University, 50-556 Wroclaw, Poland

**Keywords:** rosacea, erythema, ivermectin, oxymetazoline, azelaic acid, pulsed dye laser, microbiome, *Demodex*

## Abstract

**Background:** Rosacea is a chronic inflammatory dermatosis characterized by vascular dysregulation, immune dysfunction, neurovascular alterations, and microbial involvement. Recent advances in understanding its pathophysiology have led to the development of targeted therapeutic strategies addressing multiple disease mechanisms. This systematic review aimed to evaluate contemporary evidence regarding emerging and established treatment approaches for rosacea. **Methods:** A systematic review was conducted in accordance with the Preferred Reporting Items for Systematic Reviews and Meta-Analyses (PRISMA) guidelines. PubMed, Scopus, and Web of Science were searched for studies published between 2016 and 2025. Original human studies evaluating therapeutic interventions for rosacea were included. Study selection, data extraction, and risk-of-bias assessment were performed independently by two reviewers. Methodological quality was assessed using Joanna Briggs Institute (JBI) critical appraisal tools appropriate for each study design. **Results:** Fifteen studies involving 537 patients with rosacea and 77 controls (614 participants in total) met the eligibility criteria. Evaluated interventions included vascular-targeted therapies, topical anti-inflammatory agents, systemic and immunomodulatory treatments, and microbiome-oriented approaches. Oxymetazoline, pulsed-dye laser, platelet-rich plasma, ivermectin, azelaic acid, dapsone, sulfur preparations, and metronidazole demonstrated clinical benefits in reducing erythema, inflammatory lesions, or overall disease severity. Emerging therapies, including tofacitinib and oral ivermectin, showed promising results in refractory disease. Microbiome-related interventions, particularly *Demodex*-targeted therapies and *Helicobacter pylori* eradication, were also associated with clinical improvement. Risk-of-bias assessment identified two studies with low risk of bias, twelve with moderate risk of bias, and one study with high risk of bias. **Conclusions:** Current evidence supports a multimodal and mechanism-based approach to rosacea management, integrating vascular, inflammatory, immunological, and microbiological targets. However, the available evidence remains limited by small sample sizes, heterogeneous methodologies, short follow-up periods, and a predominance of non-randomized study designs. Large, well-designed randomized controlled trials are needed to establish optimal evidence-based treatment strategies and define the long-term efficacy and safety of emerging therapies.

## 1. Introduction

### 1.1. Clinical Overview of Rosacea

Rosacea is a long-term inflammatory skin disorder first described in the 14th century by the French surgeon Guy de Chauliac [1,2]. Previous classification categorized rosacea into four clinical subtypes. The erythematotelangiectatic subtype is characterized by frequent flushing and persistent redness in the central part of the face, sometimes accompanied by visible small blood vessels. The papulopustular subtype also involves central facial redness but, in addition, presents with transient bumps and pus-filled lesions, resembling acne. The phymatous subtype is associated with thickening of the skin, irregular surface nodules, and enlargement of affected areas, most commonly the nose, but it can also involve the chin, forehead, cheeks, or ears [3]. The ocular subtype affects the eyes and is marked by symptoms such as a foreign body sensation, burning or stinging, dryness, itching, light sensitivity, blurred vision, visible blood vessels in the eye, and swelling around it. A variant form, granulomatous rosacea, presents with firm, non-inflammatory papules or nodules that are typically uniform in size and may appear brown, yellow, or red [4]. Besides the somatic complications, treating rosacea is important because its visible and often persistent facial symptoms can significantly impair quality of life. The condition is commonly associated with reduced self-esteem, social anxiety, and emotional distress due to its impact on appearance and unpredictability of flare-ups. Effective management helps improve psychosocial well-being by reducing symptom burden and restoring confidence in social and daily interactions [5].

### 1.2. Epidemiology and Population Distribution

Rosacea is considered a relatively common chronic inflammatory skin disease; however, true global prevalence remains uncertain due to substantial methodological variability and limited data from several regions. A recent systematic review and meta-analysis of population-based studies estimated a pooled global prevalence of approximately 3.2%, although reported rates vary widely between 0.09% and 15.1% depending on population and methodology. Higher prevalence has been observed in some European countries, including Finland and Sweden, while markedly lower rates have been reported in East Asian populations such as Korea and Taiwan. This heterogeneity is largely attributed to differences in study design, diagnostic approach (clinical examination versus administrative coding), and potential underdiagnosis in individuals with darker skin types [6]. Data from a US survey (1993–2010) showed that among diagnosed patients, 3.9% were Hispanic, 2.3% Asian or Pacific Islander, and 2.0% Black. The lower reported prevalence in darker-skinned populations is likely influenced by underdiagnosis, as key symptoms like redness are more difficult to detect [7].

### 1.3. Pathophysiological Mechanisms and Disease Drivers

**Figure 1:**
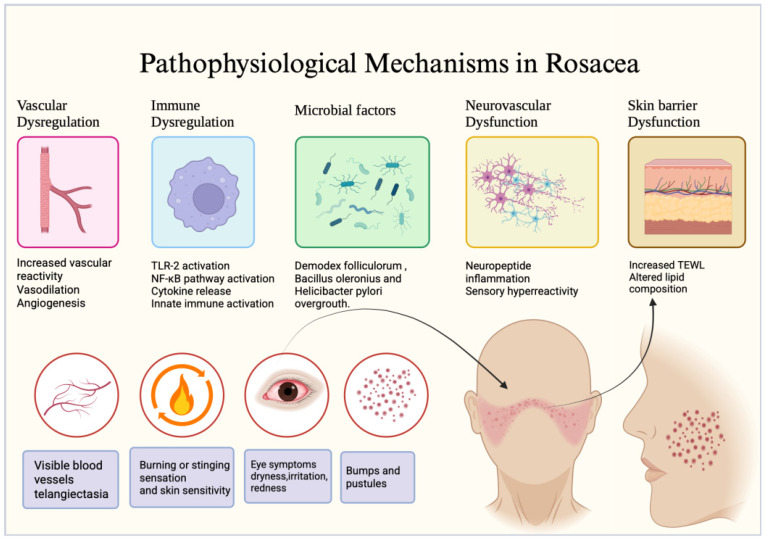
Overview of the pathophysiological mechanisms of rosacea (Created in BioRender. Andrusiewicz, A. (2026) https://BioRender.com/k0an60n accessed on 20 June 2026).

The exact etiology and pathophysiology of rosacea remain unclear. Evidence indicates that both genetic susceptibility and environmental influences contribute to its development by altering the function of the innate and adaptive immune systems [8]. Various triggers promote the release of inflammatory mediators from keratinocytes (such as cathelicidin, vascular endothelial growth factor, and endothelin-1), endothelial cells (nitric oxide), mast cells (cathelicidin and matrix metalloproteinases), and macrophages (including interferon-gamma, tumor necrosis factor, matrix metalloproteinases, and interleukin-26), along with activation of T helper type 1 (TH1) and TH17 cells [9]. These molecules drive inflammatory processes and vascular alterations associated with the condition. In addition, trigger factors can act directly on the skin’s nervous system, where neurovascular and neuro-immune neuropeptides contribute to the development of characteristic rosacea lesions [10]. The summary of pathophysiological mechanisms involved in rosacea is presented in Figure 1.

### 1.4. Molecular and Immunological Basis of Disease

Recent research has significantly expanded knowledge of the molecular mechanisms underlying rosacea, emphasizing pathways such as toll-like receptor 2 (TLR2), LL37, mammalian target of rapamycin (mTOR), interleukin-17 (IL-17), transient receptor potential vanilloid (TRPV), and the Janus kinase–signal transducer and activator of transcription (JAK-STAT) system [7]. Among these, LL37-dependent signaling, particularly through TLR2 and mTORC1, is considered a key contributor to disease pathogenesis. LL37 interacts with several intracellular signaling molecules, including ERK1/2, NF-κB, inflammasomes, CXCL8, MRGPRX2-TRPV4, and VEGF [11]. These interactions lead to the activation of immune and vascular cells such as macrophages, neutrophils, mast cells, and endothelial cells. As a result, there is increased production of proinflammatory cytokines, including TNF-α, IL-6, IL-1β, CCL5, CXCL9, and CXCL10 [12]. These mediators collectively drive immune dysregulation, inflammation, and angiogenesis observed in rosacea. Furthermore, the IL-17 and JAK-STAT signaling pathways also play important roles by enhancing inflammatory responses and promoting vascular changes associated with the condition [13].

### 1.5. Therapeutic Approaches and Pharmacological Management

This review includes the pharmacological treatment mechanisms for Rosacea as outlined in the S2k guidelines [14] and the ROSCO 2019 updated treatment algorithm [15], supplemented with additional FDA-approved therapies. While the primary focus is on pharmacological management, it is vital to also mention the role of energy-based and procedural interventions in disease control. Light-based modalities, including IPL, PDL, KTP, and Nd:YAG lasers, are effective in addressing vascular symptoms such as erythema, flushing, and telangiectasia. Ablative laser techniques (CO_2_ and Er:YAG) are mainly indicated for phymatous changes. For advanced phymatous disease, particularly rhinophyma, surgical options such as excision, dermabrasion, electrosurgery, and laser vaporization are employed, with radiofrequency ablation offering a potentially improved safety profile due to reduced thermal tissue damage. Overall, procedural therapies serve as adjuncts to pharmacological treatment, with efficacy dependent on disease subtype and operator expertise, though many techniques carry a risk of scarring [16].

#### 1.5.1. Topical Therapies

Azelaic acid has antimicrobial and anti-comedonal activity, as well as multiple anti-inflammatory effects. It suppresses the generation of reactive oxygen species and lowers levels of proinflammatory cytokines, including IL-1, IL-6, and TNF-α [17].

Studies indicate that it can also reduce UV-induced inflammatory reactions in the skin, which may explain its usefulness in conditions like rosacea that can be triggered by sunlight. Specifically, azelaic acid has been shown to inhibit UVB-induced nuclear translocation of the NF-κB p65 subunit and decrease phosphorylation of p38 mitogen- and stress-activated protein kinase [18].

In addition, it enhances peroxisome proliferator–activated receptor gamma (PPARγ) activity, a key regulator involved in controlling inflammation [19].

The precise mechanism of action of metronidazole in rosacea remains uncertain. Its therapeutic benefit is generally linked to both antimicrobial and anti-inflammatory activities. Laboratory studies have demonstrated that metronidazole can inhibit reactive oxygen species, which contribute to tissue damage during inflammation. This antioxidant effect is believed to play a major role in reducing inflammation associated with rosacea [20].

Brimonidine tartrate is a highly selective α2-adrenergic receptor agonist, with approximately 1000-fold greater selectivity for α2 receptors over α1 receptors. It produces its effects primarily through direct vasoconstriction of small arteries and veins. Research has shown that it is particularly effective in constricting human subcutaneous vessels with diameters below 200 µm [21]. In addition to its vascular effects, it also exhibits anti-inflammatory properties, including the ability to reduce edema in experimental inflammation models [22].

Ivermectin (IVM), a semi-synthetic derivative of avermectin, is used as a therapeutic option for mild to moderate inflammatory rosacea affecting the face. Its clinical benefits are linked to a combination of anti-inflammatory and antiparasitic mechanisms. It modulates inflammation by suppressing lipopolysaccharide-stimulated production of proinflammatory cytokines, including tumor necrosis factor-α (TNF-α) and interleukins IL-1β and IL-6, while simultaneously promoting the expression of the anti-inflammatory cytokine IL-10 [23]. Additionally, ivermectin targets *Demodex folliculorum* by blocking glutamate-gated chloride channels, leading to parasite paralysis and reduced mite density [24].

In the management of rosacea, sulfacetamide/sulfur is applied topically, especially in the papulopustular form. Its exact mode of action remains uncertain, but it is believed to primarily reduce inflammation. The treatment helps decrease inflammatory lesions and the redness around them, and may also lessen persistent facial erythema. It can be particularly helpful in patients who also have coexisting seborrheic dermatitis, improving overlapping symptoms [25].

In rosacea, oxymetazoline acts primarily by targeting the vascular component responsible for persistent facial erythema. As an α-adrenergic receptor agonist, it stimulates receptors on vascular smooth muscle, leading to vasoconstriction of dilated superficial blood vessels [26]. Oxymetazoline directly counteracts the chronic vasodilation driven by neurovascular dysregulation and inflammatory signaling pathways by inhibiting neutrophils and the release of pro-inflammatory cytokines. Reducing blood flow in these vessels, it helps diminish visible redness and edema. Overall, its mechanism focuses on reversing abnormal vascular responses rather than modifying the underlying inflammatory pathways [27].

#### 1.5.2. Systemic Therapies

Isotretinoin is an effective treatment for rosacea when used as prescribed, as it reduces sebaceous gland size, decreases sebum production, and normalizes keratinocyte proliferation and keratinization [28]. By modifying the follicular microenvironment, it can also lower levels of *Cutibacterium acnes* associated with the condition. In addition, it exerts anti-inflammatory and immunomodulatory effects, including reduced inflammatory cytokine release and decreased monocyte toll-like receptor 2 (TLR2) expression [29].

Tetracycline-class antibiotics, including tetracycline, doxycycline, minocycline, and sarecycline, are commonly used in dermatology for their combined antimicrobial and anti-inflammatory properties. Their therapeutic effect in rosacea is largely related to modulation of inflammation rather than direct antibacterial action [30]. These agents reduce inflammatory responses by limiting neutrophil chemotaxis, suppressing T-lymphocyte activity, and inhibiting enzymes such as phospholipase A2 and matrix metalloproteinases. They also decrease mast cell activation, neutralize reactive oxygen species, and lower the production of proinflammatory cytokines, while reducing nitric oxide synthase expression. Through these mechanisms, tetracyclines help control the inflammatory processes underlying rosacea [31].

Macrolides such as erythromycin, clarithromycin, roxithromycin, and azithromycin are antibiotics that also have important anti-inflammatory and immunomodulatory effects relevant to rosacea. In rosacea, their benefit is mainly due to suppression of inflammation rather than direct antibacterial action [32]. They reduce proinflammatory cytokines, including IL-1, IL-6, IL-8, and TNF-α, partly through inhibition of pathways such as NF-κB and activator protein-1 [33]. Macrolides also decrease neutrophil chemotaxis and infiltration, reduce leukotriene B4 production, and limit reactive oxygen species formation. In addition, they modulate immune responses by affecting T-lymphocyte activity and increasing anti-inflammatory cytokines such as IL-10, helping to control the inflammatory processes involved in rosacea [34].

Zinc is important for the development of the cell-mediated innate immune system and has antioxidant and anti-inflammatory properties relevant to rosacea. At the molecular level, it helps regulate oxidative stress and modulate immune and inflammatory pathways in the skin. Topical zinc sulfate shows consistent benefits, likely through local anti-inflammatory and antioxidant effects on the skin [35].

Cyclosporine is an immunomodulatory cyclic polypeptide used in ocular manifestations of Rosacea, especially ocular rosacea. It inhibits calcineurin-dependent T-lymphocyte activation, reducing proliferation, migration, and downstream pro-inflammatory cytokine production. This leads to decreased conjunctival inflammation, improved tear secretion, and enhanced ocular surface stability. Clinically, topical use has been associated with improved tear film parameters and meibomian gland function, reflecting its T-cell–mediated anti-inflammatory effect at the ocular surface [36].

#### 1.5.3. Off-Label and Emerging Therapies

In the context of topical therapy for rosacea, retinoids are considered a possible but not well-established option. They may provide benefits by normalizing keratinocyte turnover and exerting mild anti-inflammatory effects [37]. However, their use is often limited due to a high risk of local irritation, which can exacerbate symptoms such as erythema and skin sensitivity. Additionally, clinical evidence supporting their effectiveness in rosacea is limited and inconsistent, with a lack of standardized formulations and treatment protocols. As a result, topical retinoids are typically used off-label and with caution in carefully selected patients [38].

In rosacea, topical calcineurin inhibitors such as tacrolimus and pimecrolimus help reduce inflammation by suppressing immune system activity. They act by inhibiting calcineurin, which blocks T-cell activation and decreases the production of proinflammatory cytokines, including IL-2, IL-4, interferon-γ, and TNF-α. This leads to reduced Th1 and Th2-mediated immune responses [39]. Additionally, these agents limit activation of mast cells and neutrophils, lowering the release of inflammatory mediators. As a result, they can help control inflammatory symptoms associated with rosacea, although their use is typically off-label [40].

Botulinum toxin (BTX) may improve rosacea by targeting neurovascular mechanisms involved in facial flushing and erythema. It inhibits the release of acetylcholine from peripheral autonomic nerves, reducing cutaneous vasodilation. BTX also decreases neurogenic inflammatory mediators such as substance P and calcitonin gene-related peptide (CGRP), which are involved in promoting vasodilation and inflammation [41]. By lowering these mediators, it reduces local inflammatory activity and helps diminish redness and burning sensations. Overall, its effect in rosacea is mainly through modulation of neurovascular and neuroinflammatory signaling pathways in the skin [42].

Permethrin is a pyrethroid agent used for parasitic infections and has been explored in rosacea due to its activity against *Demodex* mites. It can also affect polymorphonuclear neutrophils (PMNs), particularly by influencing the respiratory burst that generates reactive oxygen species (ROS). Since excessive ROS and neutrophil activation contribute to inflammation in rosacea, this modulation may be beneficial. However, its overall therapeutic role in rosacea remains uncertain [43].

Benzyl benzoate works mainly by exerting toxic effects on the nervous system of ectoparasites, disrupting neuronal transmission and causing paralysis and death. At the molecular level, it interferes with normal neural signaling in mites, which underlies its antiparasitic action. In relation to rosacea, its potential relevance is linked to reducing *Demodex* mite density, which may contribute to cutaneous inflammation in susceptible individuals [44]. By lowering mite-related stimuli on the skin, it may indirectly decrease activation of innate immune responses involved in inflammatory pathways. However, it does not directly target human inflammatory or vascular mechanisms in rosacea, and its activity is primarily antiparasitic [45].

Oral β-blockers have been proposed as an off-label option for managing flushing and persistent erythema in Rosacea, conditions driven largely by neurovascular dysregulation. Their mechanism involves antagonism of sympathetic catecholamine signaling, particularly at β2-adrenergic receptors expressed in cutaneous vascular smooth muscle [46]. Blocking β2-mediated vasodilatory pathways is thought to reduce vessel dilation and promote relative vasoconstriction, thereby attenuating erythema. In addition, β-blockade may indirectly reduce flushing by dampening stress-related sympathetic activation, including anxiety- and tachycardia-associated triggers [47].

Despite the growing number of available therapeutic options, treatment selection in rosacea remains challenging because of disease heterogeneity, variable treatment responses, and the limited availability of high-quality comparative evidence. Therefore, the aim of this systematic review was to summarize and critically evaluate recent advances in rosacea treatment and to provide an updated overview of emerging therapeutic strategies.

## 2. Materials and Methods

The search was performed on 14 April 2026 in accordance with the Preferred Reporting Items for Systematic Reviews and Meta-Analyses (PRISMA) statement guidelines [48] using the databases PubMed, Scopus and Web of Science, following the PRISMA checklist (Table S1). The publication period was restricted to studies published between 2016 and 2025 to capture contemporary advances in rosacea management and emerging therapeutic approaches. Only full-text articles published in English were eligible for inclusion to ensure accurate data extraction and interpretation of study findings. The following search strategies were used:

For PubMed: (rosacea[Title/Abstract] OR “rosacea”[MeSH Terms] OR “acne rosacea”[Title/Abstract]) AND (treatment[Title/Abstract] OR therapy[Title/Abstract] OR management[Title/Abstract] OR “targeted therapy”[Title/Abstract] OR “novel therapy”[Title/Abstract] OR ivermectin[Title/Abstract] OR brimonidine[Title/Abstract] OR oxymetazoline[Title/Abstract] OR doxycycline[Title/Abstract] OR metronidazole[Title/Abstract] OR “laser therapy”[Title/Abstract]) AND Humans[MeSH Terms] AND English[lang] AND (“1 January 2016”[Date—Publication]: “31 December 2025”[Date—Publication]) AND (“journal article”[Publication Type] NOT “review”[Publication Type])—646 documents.

For Scopus: TITLE-ABS-KEY (rosacea OR “acne rosacea”) AND TITLE-ABS-KEY (treatment OR therapy OR management OR “targeted therapy” OR “novel therapy” OR ivermectin OR brimonidine OR oxymetazoline OR doxycycline OR metronidazole OR “laser therapy”) AND TITLE-ABS-KEY (human OR humans OR patients OR clinical OR trial ) AND (PUBYEAR > 2016 AND PUBYEAR < 2026) AND NOT (DOCTYPE, “re”) AND (LIMIT-TO (SUBJAREA, “PHAR”)) AND (LIMIT-TO (LANGUAGE, “English”))—369 documents.

For Web of Science: TS = (rosacea OR “acne rosacea”) AND TS = (treatment OR therapy OR management OR “targeted therapy” OR “novel therapy” OR ivermectin OR brimonidine OR oxymetazoline OR doxycycline OR metronidazole OR “laser therapy”) AND TS = (human OR humans OR patients OR clinical OR trial) AND PY = (2016–2025) NOT DT = (Review)—1065 documents.

The records were independently screened at the title, abstract, and full-text levels by two reviewers. Any disagreements regarding study eligibility were resolved through discussion and consensus. Only studies that met all predefined eligibility criteria based on the PI(E)COS framework (“Population”, “Intervention”/“Exposure”, “Comparison”, “Outcomes”, and “Study design”) [49] were included, as presented in Table 1. Data were independently extracted by two reviewers using a standardized data extraction form. Extracted variables included author, year of publication, country, study design, participant characteristics, rosacea subtype, intervention details, treatment duration, molecular targets, clinical outcomes, and principal findings. Any discrepancies in data extraction were resolved through discussion and consensus. Due to substantial heterogeneity in study design, patient populations, rosacea subtypes, therapeutic interventions, treatment duration, molecular targets, and outcome measures, statistical pooling of results was not considered appropriate. Therefore, a qualitative narrative synthesis was performed. This systematic review was not prospectively registered in PROSPERO or any other international systematic review registry. A detailed flowchart of the selection process is provided in the Results section.

Risk of bias was independently assessed by two reviewers using the Joanna Briggs Institute (JBI) Critical Appraisal Checklists appropriate for each study design. The JBI Quasi-Experimental Checklist was applied to prospective interventional studies, the JBI Case Series Checklist to retrospective studies and case series, and the JBI Randomized Controlled Trial Checklist to randomized clinical trials. Based on the overall methodological quality assessment, studies were categorized as having low, moderate, or high risk of bias. Any disagreements between reviewers were resolved through discussion and consensus. The results of the risk of bias assessment are presented in Table 2, while detailed domain-level assessments are provided in Appendix A Table A1, Table A2 and Table A3.

## 3. Results

**Figure 2:**
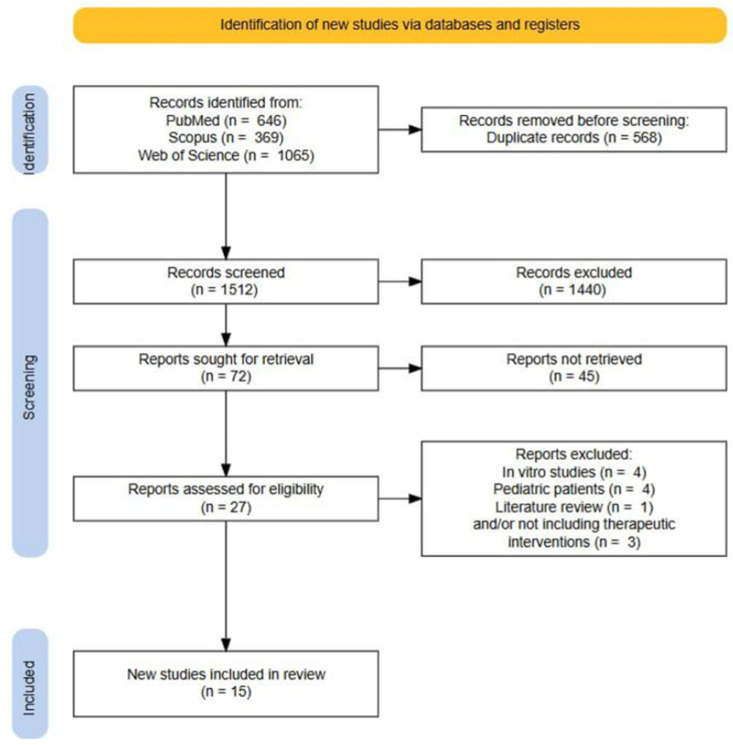
PRISMA flowchart of selected studies.

The literature search identified 2080 records, including 646 from PubMed, 369 from Scopus, and 1065 from Web of Science. After removal of 568 duplicate records, 1512 records remained for title and abstract screening. Following screening, 1440 records were excluded. The remaining 72 reports were sought for retrieval, of which 45 could not be retrieved. Consequently, 27 full-text articles were assessed for eligibility. After full-text review, 12 reports were excluded, including 4 in vitro studies, 4 studies involving pediatric patients, 1 literature review, and 3 studies that did not evaluate therapeutic interventions. Ultimately, 15 studies met the predefined eligibility criteria and were included in this systematic review. The study selection process is presented in the PRISMA flowchart (Figure 2).

A total of 15 studies, published between 2016 and 2025, were included in this systematic review. The studies were conducted across multiple geographical regions, including Iran (*n* = 2), China (*n* = 1), USA (*n* = 1), Spain (*n* = 1), Egypt (*n* = 2), Taiwan (*n* = 1), Italy (*n* = 3), Greece (*n* = 1), Japan (*n* = 1), and Turkey (*n* = 2), reflecting a broad international distribution of evidence on rosacea therapeutics. Collectively, the included studies evaluated 537 patients with rosacea and 77 controls, resulting in a total of 614 participants. The inclusion of control participants was primarily attributable to the inclusion of a split-face study and a population-based comparative cohort. The study designs comprised five clinical trials, five retrospective studies, two prospective studies, one case series, one split-face study, and one case–control study. Sample sizes ranged from small interventional studies enrolling 15–24 patients to larger clinical and retrospective studies including up to 76 participants, thereby providing a heterogeneous overview of therapeutic approaches and clinical outcomes in rosacea.

The characteristics of the included studies, including participants, molecular focus and principal findings, are summarized in Table 1.

### 3.1. Vascular, Device-Based, and Procedural Therapies

Among the 15 included studies, 5 focused on therapies targeting the vascular component of rosacea, including both pharmacological and procedural interventions. These approaches aim to reduce erythema and telangiectasia through vasoconstriction, modulation of vascular reactivity, or physical remodeling of cutaneous vessels and tissue. The inclusion of procedural techniques highlights the growing role of combination and device-based strategies in managing erythematotelangiectatic rosacea.

Sajdeh et al. [50] conducted a before–after clinical study evaluating oxymetazoline 1% cream in 15 patients with rosacea (12 females, 3 males; age range 18–50 years). Treatment efficacy was assessed using Clinician’s Erythema Assessment (CEA), Patient’s Self-Assessment (PSA), and objective skin measurements. The intervention resulted in a significant reduction in CEA score (3.46 → 2.20, *p* = 0.001) and PSA score (3.26 → 2.06, *p* = 0.001) after 4 weeks. The erythema index decreased significantly at 4 weeks (610.55 → 508.97, *p* = 0.001), and ΔE values were reduced from 6.07 to 4.50 (*p* = 0.04). Capillaroscopic evaluation showed a reduction in telangiectasia and vessel diameter. No adverse effects were reported. These findings suggest that oxymetazoline may improve erythema and vascular features in rosacea, although the lack of a control group and small sample size may limit the generalizability of the results.

Suggs et al. [52] conducted a retrospective study evaluating pulsed-dye laser (PDL) combined with oxymetazoline 1.0% cream in 31 patients with rosacea (20 females, 11 males; mean age 51 ± 13 years). Treatment efficacy was assessed using the Clinical Erythema Assessment (CEA) and a telangiectasia clearance scale. The intervention resulted in at least one-grade improvement in CEA in 55% of patients (17/31) and two-grade improvement in 13% (4/31). Additionally, 90% of patients achieved at least a two-point telangiectasia clearance (26/29), 62% achieved ≥3-point clearance (18/29), and 41% achieved ≥4-point clearance (12/29). Greater improvement was observed in patients with higher baseline erythema (CEA 3–4), with significantly higher rates of ≥1-grade (*p* = 0.021) and ≥2-grade improvement (*p* = 0.041). These findings suggest that combination therapy may improve both erythema and telangiectasia in rosacea, although the retrospective design and lack of a control group may introduce bias.

Bageorgou et al. [57] conducted a prospective unblinded clinical study evaluating topical tranexamic acid solution alone or combined with microneedling in 20 patients with erythematotelangiectatic rosacea (20 females; age range 27–65). Treatment efficacy was assessed using the Investigator Global Assessment of Rosacea Severity Score (IGA-RSS) after four sessions. Both treatment approaches resulted in clinical improvement, with a reduction of 2 points in the monotherapy group and 3 points in the combination group (*p* < 0.05), while 70% and 80% of patients, respectively, achieved at least a 2-point reduction in IGA-RSS. These findings suggest that tranexamic acid-based therapy may contribute to a reduction in erythema severity in erythematotelangiectatic rosacea, with greater improvement observed when combined with microneedling, although the small sample size and unblinded study design should be considered when interpreting the results.

Cameli et al. [56] performed a controlled clinical study evaluating a topical 18-beta glycyrrhetinic acid cream applied twice daily for 20 days in 24 patients with erythematotelangiectatic or mild papulopustular rosacea (12 patients treated, 12 controls; age range 35–70, sex not reported). Treatment efficacy was assessed using erythema index measurements and noninvasive instrumental evaluation. The intervention resulted in a significant reduction in erythema after 10 days (*p* < 0.05), which was maintained at the end of treatment, while no improvement was observed in the control group. Additionally, no significant changes in skin hydration were detected (*p* > 0.05), despite a numerical increase from baseline, and no adverse events were reported. These results indicate that topical glycyrrhetinic acid may reduce erythema in mild rosacea, although the small sample size and limited quantitative reporting may affect the strength of the conclusions.

Ghoz et al. [54] investigated the efficacy of platelet-rich plasma (PRP) injections in a split-face clinical study including 40 patients with rosacea (36 females, 4 males; mean age 43.9, range 32–65), with the opposite side receiving platelet-poor plasma as a comparator. Treatment outcomes were evaluated using the Rosacea Grading Scale (RGS) after six sessions over 12 weeks. PRP therapy led to a greater reduction in disease severity compared to control, with mean RGS decreasing from 16.1 to 6.5 versus 16.1 to 10.6 (*p* < 0.001), and all patients achieving at least good clinical improvement, including 50% with excellent (>75%) response. Additionally, PRP was associated with reduced inflammatory cell infiltration and decreased nuclear factor kappa B expression. These results indicate that PRP may contribute to clinical and histological improvement in rosacea, although the limited follow-up and within-subject design should be considered when interpreting the findings.

### 3.2. Anti-Inflammatory and Conventional Topical Therapies

A total of 6 out of 15 studies evaluated topical anti-inflammatory and conventional therapies, confirming their central role in rosacea management, particularly in inflammatory subtypes. These treatments demonstrated consistent efficacy in reducing papulopustular lesions and partially improving erythema, with generally favorable safety profiles. The diversity of agents reflects ongoing refinement within established therapeutic pathways.

Eckert et al. [53] conducted a case series evaluating topical ivermectin 1% cream in 34 patients with rosacea (29 females, 5 males; mean age not reported). Treatment efficacy was assessed using a physician-rated percentage improvement scale based on clinical and photographic evaluation. After 4 weeks, 44% of patients achieved 50–75% improvement (15/34), while 18% showed >75% improvement (6/34). After 8 weeks, 38% achieved 50–75% improvement (13/34), and 23.5% achieved >75% improvement (8/34), with an overall response observed in 85% of patients. Mild adverse effects were reported, primarily transient itching, with treatment discontinuation in one case. These results indicate that topical ivermectin could be effective in alleviating both inflammatory and vascular manifestations of rosacea; however, the case series design and the absence of standardized outcome assessments may limit the reliability of the findings due to potential bias.

Hasanbeyzade et al. [63] conducted a retrospective comparative study assessing topical azelaic acid 20% and dapsone 7.5% in 76 patients with mild-to-moderate papulopustular rosacea, including 44 patients treated with azelaic acid (41 females, 3 males; mean age 40.3 years) and 32 patients treated with dapsone (30 females, 2 males; mean age 39.7 years). Patients were followed for at least 12 weeks, and outcomes were evaluated using Investigator Global Assessment (IGA), inflammatory lesion count, and erythema scores. Both treatments were associated with significant improvements within groups (*p* < 0.001 for all), with IGA scores decreasing from 3.43 to 1.20 (~65% reduction) and from 3.50 to 1.41 (~60% reduction), respectively. Inflammatory lesions were reduced by approximately 59.6% (20.95 → 8.91) with azelaic acid and 53.0% (20.91 → 10.16) with dapsone, while erythema scores decreased by ~64.8% and ~50.5%, respectively. No significant differences were observed between the treatments in terms of efficacy (*p* > 0.05). Adverse events occurred more frequently with azelaic acid (34.1% vs. 12.5%, *p* = 0.032). These results suggest comparable clinical effectiveness between the two therapies, with a potentially more favorable tolerability profile for dapsone.

Gökşin et al. [64] carried out a prospective clinical study investigating topical dapsone 5% gel in 35 patients with erythematotelangiectatic rosacea (22 females, 13 males; age 38, range 19–62). Treatment efficacy was assessed using Investigator Global Assessment (IGA) over 12 weeks. The therapy led to a reduction in severe disease (IGA ≥ 3) from 37.2% at baseline to 0% at week 12 (*p* < 0.001), with an overall treatment success rate of 62.9%, alongside improvement in patient-reported symptoms reflected by a decrease in VAS score from 7 (5 to 9) to 4 (2–6) (*p* < 0.001) and enhanced quality of life with DLQI reduction from 8 (6 to 14) to 4 (2–9) (*p* < 0.001). These findings suggest that topical dapsone may confer meaningful clinical benefit in mitigating disease severity and alleviating symptom burden in erythematotelangiectatic rosacea; however, the lack of a control group may predispose the results to potential bias.

Nobeyama et al. [59] analyzed real-world clinical data to assess the effectiveness of topical sulfur preparation (TSP) and topical metronidazole preparation (TMP) in 47 patients with rosacea (22 treated with TSP, including 10 ETR and 12 PPR; 25 treated with TMP, including 12 ETR and 13 PPR; mean age 46.2 vs. 48.1 years). Treatment efficacy was evaluated using Investigator Global Assessment (IGA) and Visual Analog Scale (VAS) over 8 weeks. Both treatments were associated with significant improvement in disease severity and symptom scores in ETR and PPR patients (*p* < 0.05), with no significant differences between TSP and TMP in overall clinical response. Additionally, *Demodex* positivity decreased from 81.2% to 28.6% in the TSP group (*p* < 0.001) and from 83.3% to 40.0% in the TMP group (*p* = 0.003). Overall, the findings support comparable effectiveness of sulfur and metronidazole preparations in routine clinical management of rosacea, while the retrospective design should be taken into account.

Dall’Oglio et al. [62] conducted a multicentre, prospective clinical study evaluating a novel topical formulation containing azelaic acid 15% combined with dihydroavenanthramide D 1% in 45 patients with inflammatory rosacea (34 females, 10 males; mean age 46.1 years). Patients applied the treatment twice daily for 8 weeks, and efficacy was assessed using Investigator Global Assessment (IGA), inflammatory lesion count, and erythema severity measured by erythema-directed digital photography. The intervention resulted in a reduction in IGA score (3 → 1, *p* < 0.001), accompanied by an approximately 87.5% decrease in inflammatory lesions (median 8 → 1, *p* < 0.001) and improvement in erythema severity (2 → 1, *p* < 0.001). The treatment was well tolerated, with only one mild adverse event reported. These results indicate that this formulation may reduce both inflammatory and vascular components of rosacea with good tolerability.

Dall’Oglio et al. [61] carried out a prospective open-label study assessing the correlation between clinical and instrumental evaluation of erythema in rosacea during topical therapy. The study involved 20 patients (17 females, 3 males; mean age 46.9 years) with mild to moderate inflammatory rosacea treated with ivermectin 1% cream once daily for 8 weeks. Erythema severity was evaluated at baseline and at weeks 2, 4, 6, and 8 using clinician erythema assessment (CEA), erythema-directed digital photography (EDDP), and colorimeter (COL). At baseline, a significant concordance and correlation between EDDP and COL were observed (κ = 0.578; r = 0.637), while no agreement with clinical assessment was noted. During treatment, concordance progressively increased, reaching very high agreement at week 8 (e.g., κ ≈ 0.927 for CEA–EDDP and κ ≈ 0.925 for CEA–COL). Instrumental methods detected early erythema reduction already at week 2, which was not captured clinically. These findings indicate that EDDP and colorimetry provide a more sensitive and objective assessment of erythema, particularly in early treatment phases, while clinical and instrumental evaluations become more aligned as therapeutic response progresses.

### 3.3. Systemic and Immunomodulatory Therapies

Two of the included studies investigated systemic and immunomodulatory approaches, representing more advanced treatment strategies for rosacea. These therapies target underlying immune dysregulation and may be particularly beneficial in patients with refractory or more severe disease. However, current evidence is limited by small sample sizes and non-randomized study designs.

Sun et al. [51] conducted a retrospective case series evaluating oral tofacitinib in 21 patients with rosacea (19 females, 2 males; mean age 35.5 years, range 24–49). Treatment efficacy was assessed using the Investigator’s Global Assessment (IGA) and patient-reported outcomes. The intervention resulted in treatment success (IGA ≤ 1) in 71.4% of patients (15/21), with a mean reduction in IGA score of 2.24 points (*p* < 0.0001). Additionally, 90.5% of patients achieved an improvement of ≥2 points in IGA score, and 81.0% reported satisfaction with treatment. Limited adverse events were observed in 2 patients. The results imply that tofacitinib has the potential to ameliorate the clinical manifestations of rosacea; nevertheless, the retrospective nature of the study, together with the absence of a comparator group, may limit the robustness of these observations due to inherent bias.

Sharara et al. [58] conducted a single-arm clinical study evaluating a single oral dose of ivermectin (250 μg/kg) in 45 patients with rosacea (31 females, 14 males; mean age 27.8, range 19–37). Treatment efficacy was assessed using Investigator Global Assessment (IGA) over a 3-month follow-up period. The intervention resulted in a significant overall improvement in disease severity (*p* < 0.001), with the proportion of patients achieving clear skin increasing from 0% at baseline to 33.3% after treatment, while moderate cases decreased from 28.9% to 4.4%. Additionally, *Demodex* density showed a highly significant reduction following treatment (*p* < 0.001), and 46.7% of patients reported marked clinical improvement. These results indicate that single-dose oral ivermectin may improve clinical severity and reduce *Demodex* burden in rosacea, although the absence of a control group may limit the strength of the conclusions.

### 3.4. Microbiome- and Etiology-Oriented Therapies

Two studies focused on etiological and microbiome-related aspects of rosacea, emphasizing the role of microbial factors in disease pathogenesis. These approaches aim to reduce microbial burden or eliminate potential triggers, which may lead to clinical improvement. The findings support the relevance of the skin–microbiome axis, although causality remains to be fully established.

Huang et al. [55] conducted a retrospective study evaluating topical ivermectin 1% cream alone or combined with oral carvedilol in 36 patients with rosacea and persistent facial erythema with high *Demodex* density (ivermectin group *n* = 14; ivermectin–carvedilol group *n* = 22; age and sex matched, not reported). Treatment efficacy was assessed using Clinician’s Erythema Assessment (CEA). Both regimens resulted in significant improvement in erythema, with CEA scores decreasing from 1.9 ± 0.7 to 1.1 ± 0.3 and from 2.2 ± 0.4 to 1.2 ± 0.4 (both *p* < 0.01), and treatment success (CEA ≤ 1) was achieved in 83% of patients. Additionally, *Demodex* density was reduced from 120 ± 25.0 to 40.7 ± 15.2 mites/cm^2^ and from 115 ± 23.0 to 28.0 ± 10.0 mites/cm^2^ (both *p* < 0.01), with a correlation between reduction in erythema and mite density (rho = 0.50, *p* = 0.002). These results suggest that ivermectin-based regimens may be associated with concurrent reductions in erythema severity and *Demodex* density in rosacea, although the retrospective design and lack of detailed demographic data should be considered when interpreting the findings.

Aghaei et al. [60] performed a randomized controlled study evaluating the effect of *Helicobacter pylori* eradication therapy in 60 patients with rosacea (48 females, 12 males; median age 48), including 30 patients receiving standard rosacea treatment plus a 2-week triple eradication regimen (clarithromycin, metronidazole, and pantoprazole) and 30 receiving standard treatment alone, alongside 65 healthy controls. Treatment efficacy was assessed using the Duluth rosacea severity score over 60 days. The intervention resulted in a greater reduction in disease severity in the eradication group compared to controls, with mean scores decreasing from 9.32 to 2.39 versus 9.2 to 5.35 (*p* = 0.001). Moreover, *H. pylori* seropositivity was significantly higher in rosacea patients than in healthy controls (100% vs. 6.5%). Overall, these findings indicate that eradication therapy may contribute to clinical improvement in rosacea, although the open-label design should be considered when interpreting the results.

### 3.5. Risk of Bias Assessment

Risk of bias assessment was performed using the Joanna Briggs Institute (JBI) Critical Appraisal Checklists appropriate for each study design. Overall, two studies were classified as having a low risk of bias, twelve studies as having a moderate risk of bias, and one study as having a high risk of bias. The detailed results of the methodological quality assessment are presented in Table 3.

## 4. Discussion

**Figure 3:**
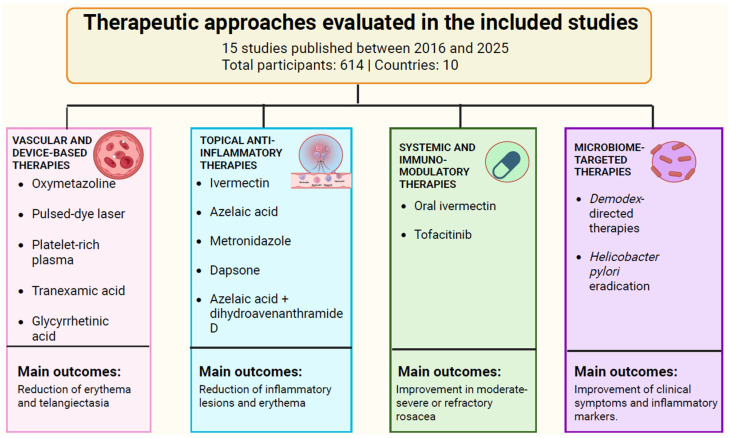
Overview of therapeutic approaches evaluated in the included studies (Created in BioRender. Andrusiewicz, A. (2026) https://BioRender.com/j4hf8xd accessed on 20 June 2026).

This systematic review provides a comprehensive overview of recent advances in rosacea treatment, reflecting the multifactorial nature of the disease and the increasing shift toward mechanism-based therapeutic strategies. The included studies were categorized into four main groups: vascular and procedural therapies, topical anti-inflammatory treatments, systemic approaches, and microbiome-oriented interventions, highlighting the diversity of current treatment modalities. Available treatments are schematically presented in Figure 3.

Therapies targeting the vascular component of rosacea play a crucial role in the management of persistent erythema and telangiectasia. Topical vasoconstrictive agents, such as oxymetazoline, demonstrated significant efficacy in reducing erythema severity and improving both clinician- and patient-reported outcomes [50]. Enhanced clinical benefits were observed with combination approaches, particularly pulsed-dye laser therapy combined with oxymetazoline, which showed greater effectiveness in patients with more severe baseline erythema [52]. Additional strategies, including tranexamic acid and glycyrrhetinic acid, also demonstrated clinically meaningful improvements in erythema [56,57]. Furthermore, procedural approaches such as platelet-rich plasma (PRP) showed potential in improving both clinical and histological parameters, suggesting a role in tissue remodeling and inflammatory modulation [54]. While vascular- and device-oriented therapies primarily address the erythematous component of rosacea, they are often complemented by treatments targeting inflammatory lesions.

Topical anti-inflammatory therapies remain the cornerstone of rosacea management, particularly in papulopustular subtypes. Ivermectin demonstrated high response rates and sustained clinical improvement, likely due to its combined anti-inflammatory and anti-parasitic effects [53]. Similarly, azelaic acid and dapsone showed comparable efficacy in reducing inflammatory lesions and erythema, although differences in tolerability may influence treatment selection [63]. Dapsone monotherapy also resulted in significant improvements in disease severity and quality of life [64]. In addition, real-world evidence confirmed the effectiveness of traditional agents such as sulfur and metronidazole [59]. Notably, a novel formulation combining azelaic acid with dihydroavenanthramide demonstrated substantial reductions in both inflammatory lesions and erythema [62]. Moreover, advancements in outcome assessment, such as erythema-directed digital photography and colorimetry, allow for more sensitive detection of treatment response [61]. Although topical therapies remain the first-line treatment, not all patients achieve satisfactory disease control.

In such cases, systemic and immunomodulatory therapies may provide an alternative approach. The use of the Janus kinase inhibitor tofacitinib was associated with significant clinical improvement and high patient satisfaction, indicating the potential of targeting immune signaling pathways [51]. Similarly, oral ivermectin demonstrated efficacy in reducing both disease severity and *Demodex* density, supporting its role in selected patients [58]. However, these findings are limited by the lack of randomized controlled trials and small sample sizes.

Beyond immune modulation, increasing attention has been directed toward microbiological and etiological factors in rosacea. Targeting *Demodex* mites was associated with improvements in erythema and clinical severity, suggesting a link between microbial load and disease activity [55]. In addition, eradication of *Helicobacter pylori* infection resulted in greater clinical improvement compared to standard therapy alone [60]. These findings support the concept that microbiological triggers may contribute to disease pathogenesis, although a direct causal relationship remains to be definitively established.

The heterogeneity of rosacea and the diversity of therapeutic responses observed across studies highlight the importance of individualized treatment selection. Patients with persistent erythema and telangiectasia may benefit primarily from vascular-targeted therapies, whereas those with papulopustular rosacea may respond better to topical anti-inflammatory agents. Systemic and immunomodulatory therapies may be considered in refractory or more severe cases, while microbiome-oriented approaches may be particularly relevant in patients with increased *Demodex* density. Future studies should further explore phenotype-driven treatment strategies to optimize therapeutic outcomes.

Interestingly, recurring research patterns were observed across different studies. Some authors contributed multiple investigations addressing complementary aspects of rosacea management, such as both therapeutic efficacy and outcome assessment [61,62]. At the same time, consistent methodological limitations were noted across studies, including small sample sizes, retrospective designs, and lack of control groups [51,52,55]. This consistency suggests that these limitations reflect broader challenges within the field rather than isolated study weaknesses.

Some limitations of the available evidence have to be acknowledged. Despite the promising results, the overall quality of evidence remains moderate to low. The heterogeneity of study designs, variability in outcome measures, short follow-up periods, and predominance of non-randomized studies limit the generalizability of the findings. Most included studies were retrospective, uncontrolled, or small prospective investigations, which should be considered when interpreting the reported treatment effects. Furthermore, potential publication bias cannot be excluded, particularly because gray literature, conference proceedings, and trial registries were not systematically searched. The interpretation of this review should also take into account two methodological constraints. First, the inclusion criteria were limited to English-language publications, which may have introduced language bias and may have resulted in the omission of relevant studies published in other languages. Second, the review protocol was not registered in a public registry, which may reduce transparency regarding whether all methodological decisions were predefined before the review was conducted.

In conclusion, current advances in rosacea treatment emphasize a transition toward targeted and multimodal approaches that address vascular, inflammatory, immunological, and microbiological aspects of the disease. While emerging therapies show considerable potential, particularly in refractory cases, their role in routine clinical practice remains to be fully established. Future research should focus on large-scale, well-designed randomized controlled trials with standardized outcome measures to better define optimal treatment strategies.

## 5. Conclusions

This systematic review demonstrates that rosacea is a multifactorial inflammatory and vascular disorder driven by the interplay of innate immune dysregulation, neurovascular instability, microbial factors, and oxidative stress. The available clinical evidence indicates that therapeutic efficacy is mediated through modulation of complementary pathogenic pathways, including inflammatory signaling (TLR2/NF-κB), cytokine activity, vascular reactivity, and *Demodex*-associated mechanisms.

Across the included studies, both topical and systemic pharmacological therapies, as well as device-based interventions, were associated with clinical improvement in erythema, inflammatory lesions, and overall disease severity, although treatment responses varied depending on rosacea subtype and therapeutic modality. Emerging data further suggest potential benefits of combination approaches and microbiome-targeted strategies in selected patient populations.

However, the current evidence is limited by heterogeneity in study design, small sample sizes, short follow-up periods, and a lack of standardized outcome measures, which restrict the comparability and generalizability of findings.

Collectively, these results support a multimodal, mechanism-informed approach to rosacea management and highlight the need for large-scale, well-designed randomized controlled trials with standardized endpoints to establish optimized evidence-based treatment strategies.

## Figures and Tables

**Table 1:** Characteristics of included studies.

Author, Year and Country	Sajdeh et al. [50] 2025, Iran	Sun et al. [51] 2022, China	Suggs et al. [52] 2020, USA	Eckert et al. [53] 2016, Spain	Ghoz et al. [54] 2020, Egypt
Study design	clinical trial	retrospective study	retrospective study	case series	split-face study
Participants	15 patients with rosacea	21 patients with rosacea	31 patients with rosacea	34 patients with rosacea	40 patients with rosacea
Rosacea Subtype	ETR	12 ETR, 9 PPR	ETR	11 ET, 23 PPR	24 PPR, 16 ETR
Dose, Regimen and Duration	topical, twice daily 4 weeks	oral, 5 mg BID → reduced to 5 mg QD/QOD; 2–26 weeks (mean 7 weeks)	topical, once daily (~4 months) + PDL (1–4 sessions, ~2 avg, 4–6 week intervals)	topical, QD, 8 weeks	PRP injections every 2 weeks, 3 months (6 sessions)
Treatment	oxymetazoline 1% cream	tofacitinib	oxymetazoline 1% cream, PDL	ivermectin 1% cream	platelet-rich plasma (PRP)
Target Pathway/Molecule	α1A-adrenergic receptor	JAK1/3, JAK–STAT pathway (IL-2, IL-4, IL-6, IL-9, IL-21)	oxyhemoglobin (laser target), α-adrenergic receptor	*Demodex folliculorum*; TLR2, LL-37, KLK5	NF-κB; growth factors (PDGF, TGF-β, VEGF)
Clinical Outcomes	CEA ↓ PSA ↓ erythema ↓ telangiectasia ↓	IGA ↓ erythema ↓	CEA ↓ telangiectasia ↓	Inflammatory lesions ↓ erythema ↓ sustained response	RGS ↓ inflammation ↓ NF-κB ↓ recurrence ↓ pain, erythema, edema ↓
Mechanism of Action	vasoconstriction, reduction in cutaneous blood flow	JAK1/3 inhibition → suppression of pro-inflammatory cytokine signaling	selective photothermolysis + vasoconstriction	anti-parasitic effect against *Demodex* mites + modulation of cutaneous inflammation	anti-inflammatory + immunomodulation, tissue repair via growth factors
**Author, Year and Country**	**Huang et al.** [55] **2021, Taiwan**	**Cameli et al.** [56] **2020, Italy**	**Bageorgou et al.** [57] **2019, Greece**	**Sharara et al.** [58] **2024, Egypt**	**Nobeyama et al.** [59] **2023, Japan**
Study design	retrospective study	case–control study	prospective study	clinical trial	retrospective study
Participants	36 patients with rosacea	24 patients (12 with rosacea, 12 controls)	20 patients with rosacea	45 patients with rosacea	47 patients with rosacea
Rosacea Subtype	16 PPR, 23 ETR	ETR, PPR	ETR	28 PPR, 17 ETR	25 PPR, 22 ETR
Dose, Regimen and Duration	topical QD ± carvedilol oral 6.25 mg BID; 1–12 weeks	topical, BID, 20 days	topical, every 15 days × 4 sessions (±microneedling)	oral, 250 µg/kg single dose	topical, BID, 8 weeks
Treatment	ivermectin 1% cream, carvedilol	18β-glycyrrhetinic acid	tranexamic acid	ivermectin	sulfur preparation or metronidazole
Target Pathway/Molecule	*Demodex*, TLR, inflammatory pathways	ROS; inflammatory pathways	plasmin, VEGF, endothelial activation	*Demodex*, TLR2, IL-8, IL-1β, TNF-α	*Demodex*, TLR2, IL-8, TNF-α, neurovascular pathways
Clinical Outcomes	CEA ↓ erythema ↓ *Demodex* density ↓	erythema ↓ erythema index ↓ lesions ↓ hydration ↔	IGA-RSS ↓ erythema ↓ telangiectasia ↓ DLQI ↓	*Demodex* density↓ IGA↓ relapse rate ↓ patient satisfaction ↑	IGA score ↓ VAS symptoms: itching ↓ burning ↓ flushing ↓ hypersensitivity ↓ *Demodex* density ↓
Mechanism of Action	anti-parasitic (*Demodex* ↓) + anti-inflammatory → ↓ TLR2 activation, carvedilol → vasoconstriction	anti-inflammatory + antioxidant → ↓ ROS-mediated inflammation	anti-fibrinolytic → ↓ angiogenesis + vascular permeability	anti-parasitic (*Demodex* ↓) + anti-inflammatory → ↓ TLR2 activation → ↓ innate immune response → ↓ cutaneous inflammation	anti-parasitic (*Demodex* ↓) + anti-inflammatory → ↓ neurovascular dysregulation
**Author, Year and Country**	**Aghaei et al.** [60] **2023, Iran**	**Dall’Oglio et al.** [61] **2021, Italy**	**Dall’Oglio et al.** [62] **2021, Italy**	**Hasanbeyzade et al.** [63] **2025, Turkey**	**Gökşin et al.** [64] **2024, Turkey**
Study design	clinical trial	clinical trial	clinical trial	retrospective study	prospective study
Participants	60 patients with rosacea (+ 65 healthy controls)	20 patients with rosacea	45 patients with rosacea (44 completed)	76 patients with rosacea	35 patients with rosacea
Rosacea Subtype	mixed (active/inactive; not subtype-specific)	mild/moderate inflammatory rosacea	mild/moderate inflammatory rosacea	PPR	ETR
Dose, Regimen and Duration	oral clarithromycin 500 mg BID + metronidazole 500 mg BID + pantoprazole 40 mg QD → 60 d	topical, QD, 8 weeks	topical, azelaic acid 15% + dihydroavenanthramide D 1%, BID, 8 weeks	topical, once daily, ≥12 weeks	topical, BID, 12 weeks
Treatment	clarithromycin, metronidazole, pantoprazole	ivermectin 1% cream	azelaic acid 15%, dihydroavenanthramide D 1%	azelaic acid 20%, dapsone 7.5%	dapsone 5% gel
Target Pathway/Molecule	*H. pylori*, TLR, TNF-α, IL-1β, ROS, NO, MMPs VEGF, neurovascular dysregulation	N\A	N\A	reactive oxygen species, kallikrein-5/cathelicidin pathway (azelaic acid); neutrophil-mediated inflammation (dapsone)	VEGF, inflammation
Clinical Outcomes	IgG, IgM ↓ disease severity ↓ (Duluth score) TLR signaling ↓	erythema ↓ (CEA, EDDP, COL) *Demodex* density ↓ ↑ concordance: clinical vs. instrumental assessment	IGA score ↓ inflammatory lesions count ↓ erythema (EDDP score) → significant improvement	IGA ↓ inflammatory lesion count ↓ erythema ↓ no significant difference between groups, fewer adverse effects with dapsone	IGA ↓ VAS ↓ DLQI high treatment success, mild adverse effects
Mechanism of Action	anti-bacterial (*H. pylori* ↓) + anti-inflammatory → ↓ endotoxin-driven immune activation → inflammation + vascular dysregulation	anti-parasitic (*Demodex* ↓, indirect) + anti-inflammatory → ↓ erythema	anti-inflammatory + antioxidant + antimicrobial → ↓ cytokine signaling → ↓ papules/pustules + erythema	anti-inflammatory + antioxidant activity (azelaic acid) → ↓ neutrophil activity + inflammatory pathways (dapsone)	anti-inflammatory → ↓ inflammation + ↓ angiogenesis

BID—bis in die; CEA—Clinician Erythema Assessment; COL—Colorimetry; DLQI—Dermatology Life Quality Index; EDDP—Erythema Directed Digital Photography; ET—erythematotelangiectatic; ETR—erythematotelangiectatic rosacea; *H. pylori*—*Helicobacter pylori*; IGA—Investigator Global Assessment; IGA-RSS—Investigator Global Assessment–Rosacea Severity Score; IgG/IgM—immunoglobulin G/immunoglobulin M; IL-1β/2/4/6/8/9/21—interleukin 1 beta/2/4/6/8/9/21; JAK—Janus kinase; JAK1/3—Janus kinase 1 and 3; KLK5—kallikrein-related peptidase 5; LL-37—cathelicidin LL-37; MMPs—matrix metalloproteinases; NF-κB—nuclear factor kappa B; NO—nitric oxide; PDGF—platelet-derived growth factor; PDL—pulsed dye laser; PPR—papulopustular rosacea; PRP—platelet-rich plasma; PSA—Patient Self-Assessment; QD/QOD—quaque die/quaque altera die; RGS—Rosacea Global Severity; ROS—reactive oxygen species; TGF-β—transforming growth factor beta; TLR/TLR2—Toll-like receptor/Toll-like receptor 2; TNF-α—tumor necrosis factor alpha; VAS—Visual Analog Scale; VEGF—vascular endothelial growth factor.

**Table 2:** Eligibility criteria according to PI(E)COS framework.

Parameter	Inclusion Criteria	Exclusion Criteria
Population	Human participants diagnosed with rosacea (any clinical subtype) Adults (≥18 years).	Participants without a confirmed diagnosis of rosacea. Studies including pediatric populations (<18 years).
Intervention/Exposure	Pharmacological and non-pharmacological therapeutic interventions for rosacea (e.g., topical and systemic treatments, laser and light-based therapies, combination therapies).	Studies not evaluating a therapeutic intervention. Studies focused exclusively on pathophysiology without treatment assessment. Cosmetic or skincare products without a defined therapeutic indication.
Comparison	Placebo, no treatment, standard of care, or alternative therapeutic interventions. Within-subject (pre–post) comparisons when a control group is not available.	Studies without any comparator or baseline assessment preventing evaluation of the treatment effect.
Outcomes	Clinically relevant efficacy and safety outcomes, including reduction in erythema, inflammatory lesion count, telangiectasia, and patient-reported outcomes, assessed using validated or clearly defined measures.	Studies not reporting clinically meaningful outcomes. Outcomes not allowing assessment of treatment efficacy or safety.
Study design	Original research articles conducted in human subjects, including randomized controlled trials (RCTs), non-randomized clinical trials, cohort studies, case–control studies, case series, and prospective or retrospective observational studies evaluating therapeutic interventions for rosacea.	Case reports, literature reviews, systematic reviews, meta-analyses, editorials, commentaries, letters to the editor, conference abstracts, non-English full-text publications, studies published before 2016, and studies without accessible full text.

**Table 3:** Risk of bias assessment of included studies using Joanna Briggs Institute (JBI) critical appraisal tools.

Study	Study Design	JBI Tool	Risk of Bias
Sajdeh et al. [50], 2025	Quasi-experimental clinical trial	JBI Quasi-Experimental Checklist	Moderate
Sun et al. [51], 2022	Retrospective case series	JBI Case Series Checklist	Moderate
Suggs et al. [52], 2020	Retrospective study	JBI Case Series Checklist	Moderate
Cameli et al. [56], 2020	Controlled clinical trial	JBI Quasi-Experimental Checklist	Low
Ghoz et al. [54], 2021	Split-face quasi-experimental study	JBI Quasi-Experimental Checklist	Low
Eckert et al. [53], 2016	Case series	JBI Case Series Checklist	High
Huang et al. [55], 2021	Retrospective case series	JBI Case Series Checklist	Moderate
Bageorgou et al. [57], 2019	Prospective comparative study	JBI Quasi-Experimental Checklist	Moderate
Sharara et al. [58], 2024	Single-arm clinical trial	JBI Quasi-Experimental Checklist	Moderate
Nobeyama et al. [59], 2023	Retrospective observational study	JBI Case Series Checklist	Moderate
Hasanbeyzade et al. [63], 2025	Retrospective comparative study	JBI Case Series Checklist	Moderate
Gökşin et al. [64], 2024	Prospective single-arm clinical study	JBI Quasi-Experimental Checklist	Moderate
Dall’Oglio et al. [62], 2021	Multicentre prospective clinical trial	JBI Quasi-Experimental Checklist	Moderate
Dall’Oglio et al. [61], 2021	Open-label prospective clinical trial	JBI Quasi-Experimental Checklist	Moderate
Aghaei et al. [60], 2023	Randomized controlled clinical trial	JBI Randomized Controlled Trial Checklist	Moderate

## Data Availability

The original contributions presented in this study are included in the article and Supplementary Material. Further inquiries can be directed to the corresponding author.

## Supplementary Materials

The following supporting information can be downloaded at: https://www.mdpi.com/article/10.3390/ph19070982/s1, Table S1: PRISMA 2020 Checklist.

## Abbreviations

The following abbreviations are used in this manuscript:

BTX	botulinum toxin
CCL5	C-C motif chemokine ligand 5
CEA	Clinician’s Erythema Assessment
CGRP	calcitonin gene-related peptide
COL	colorimeter/clinical color measurement
CXCL8	C-X-C motif chemokine ligand 8
CXCL9	C-X-C motif chemokine ligand 9
CXCL10	C-X-C motif chemokine ligand 10
DLQI	Dermatology Life Quality Index
EDDP	erythema-directed digital photography
EGF	epidermal growth factor
ERK1/2	extracellular signal-regulated kinases 1 and 2
ET	erythematotelangiectatic rosacea (subtype)
ETR	erythematotelangiectatic rosacea
*H. pylori*	*Helicobacter pylori*
IFN-γ	interferon gamma
IGA	Investigator Global Assessment
IGA-RSS	Investigator Global Assessment–Rosacea Severity Score
IL-1	interleukin-1
IL-1β	interleukin-1 beta
IL-10	interleukin-10
IL-17	interleukin-17
IL-2	interleukin-2
IL-21	interleukin-21
IL-26	interleukin-26
IL-4	interleukin-4
IL-6	interleukin-6
IL-8	interleukin-8
IL-9	interleukin-9
IVM	ivermectin
JAK	Janus kinase
JAK-STAT	Janus kinase–signal transducer and activator of transcription pathway
JAK1/3	Janus kinase 1 and 3
KLK5	kallikrein-related peptidase 5
LL-37	cathelicidin antimicrobial peptide LL-37
MAPK	mitogen-activated protein kinase
MMPs	matrix metalloproteinases
MRGPRX2	Mas-related G-protein coupled receptor X2
mTOR	mammalian Target Of Rapamycin
mTORC1	mechanistic Target Of Rapamycin Complex 1
N/A	Not Applicable/Not Available
NF-κB	nuclear factor kappa B
NO	nitric oxide
PDGF	platelet-derived growth factor
PDL	pulsed dye laser
PMN(s)	polymorphonuclear neutrophil(s)
PPARγ	Peroxisome Proliferator-Activated Receptor gamma
PPR	papulopustular rosacea
PRP	platelet-rich plasma
PSA	Patient’s Self-Assessment
QD	once daily
QOD	every other day
RGS	Rosacea Grading Scale
ROS	reactive oxygen species
STAT	signal transducer and activator of transcription
TGF-β	transforming growth factor beta
TH1	T helper 1 lymphocytes
TH17	T helper 17 lymphocytes
TH2	T helper 2 lymphocytes
TLR	toll-like receptor
TLR2	toll-like receptor 2
TMP	topical metronidazole preparation
TNF-α	tumor necrosis factor alpha
TRPV	transient receptor potential vanilloid
TRPV4	transient receptor potential vanilloid 4
TSP	topical sulfur preparation
VAS	Visual Analog Scale
VEGF	vascular endothelial growth factor
ΔE	color difference value (Delta E)

## Appendix A

**Table A1:** Risk of bias assessment—JBI Quasi-Experimental Checklist.

Study	Q1	Q2	Q3	Q4	Q5	Q6	Q7	Q8	Q9	Overall
Sajdeh	Y	Y	Y	N	Y	Y	Y	Y	Y	Moderate
Cameli	Y	Y	Y	Y	Y	U	Y	Y	Y	Low
Ghoz	Y	Y	Y	Y	Y	U	Y	Y	Y	Low
Bageorgou	Y	Y	Y	N	Y	Y	Y	Y	Y	Moderate
Sharara	Y	Y	Y	N	Y	Y	Y	Y	Y	Moderate
Gökşin	Y	Y	Y	N	Y	Y	Y	Y	Y	Moderate
Dall’Oglio AzA + DHD	Y	Y	Y	N	Y	Y	Y	Y	Y	Moderate
Dall’Oglio IVM	Y	Y	Y	N	Y	Y	Y	Y	Y	Moderate

**Table A2:** Risk of bias assessment—JBI Case Series Checklist.

Study	Q1	Q2	Q3	Q4	Q5	Q6	Q7	Q8	Q9	Q10	Overall
Sun	Y	Y	Y	U	U	Y	Y	Y	Y	Y	Moderate
Suggs	Y	Y	Y	U	U	Y	Y	Y	Y	Y	Moderate
Eckert	Y	Y	Y	U	U	Y	Y	Y	Y	N	High
Huang	Y	Y	Y	U	U	Y	Y	Y	Y	Y	Moderate
Nobeyama	Y	Y	Y	U	U	Y	Y	Y	Y	Y	Moderate
Hasanbeyzade	Y	Y	Y	U	U	Y	Y	Y	Y	Y	Moderate

**Table A3:** Risk of bias assessment—JBI Randomized controlled Trials Checklist.

Study	Q1	Q2	Q3	Q4	Q5	Q6	Q7	Q8	Q9	Q10	Q11	Q12	Q13	Overall
Aghaei	Y	Y	N	N	Y	Y	Y	Y	Y	Y	Y	Y	Y	Moderate

## References

[1] Kim H.S. (2020). Microbiota in Rosacea. Am. J. Clin. Dermatol..

[2] Thiboutot D., Anderson R., Cook-Bolden F., Draelos Z., Gallo R.L., Granstein R.D., Kang S., Macsai M., Gold L.S., Tan J. (2020). Standard Management Options for Rosacea: The 2019 Update by the National Rosacea Society Expert Committee. J. Am. Acad. Dermatol..

[3] van Zuuren E.J., Arents B.W.M., van der Linden M.M.D., Vermeulen S., Fedorowicz Z., Tan J. (2021). Rosacea: New Concepts in Classification and Treatment. Am. J. Clin. Dermatol..

[4] Oge’ L.K., Muncie H.L., Phillips-Savoy A.R. (2015). Rosacea: Diagnosis and Treatment. Am. Fam. Physician.

[5] Yang F., Zhang Q., Song D., Liu X., Wang L., Jiang X. (2022). A Cross-Sectional Study on the Relationship Between Rosacea Severity and Quality of Life or Psychological State. Clin. Cosmet. Investig. Dermatol..

[6] Geng R.S.Q., Mohsen S., Bestavros S., Ramsay K., Sibbald C. (2025). Prevalence of Rosacea: A Systematic Review and Meta-Analysis of Population-Based Studies. JAAD Rev..

[7] Geng R.S.Q., Bourkas A.N., Mufti A., Sibbald R.G. (2024). Rosacea: Pathogenesis and Therapeutic Correlates. J. Cutan. Med. Surg..

[8] Korsing S., Stieler K., Pleyer U., Blume-Peytavi U., Vogt A. (2025). Rosacea in Childhood and Adolescence: A Review. J. Dtsch. Dermatol. Ges..

[9] Buddenkotte J., Steinhoff M. (2018). Recent Advances in Understanding and Managing Rosacea. F1000Research.

[10] Wang X., Shi H., Li X., Feng Y. (2025). Macrophages in Rosacea: Pathogenesis and Therapeutic Potential. Front. Immunol..

[11] Yang F., Wang L., Song D., Zhang L., Wang X., Du D., Jiang X. (2024). Signaling Pathways and Targeted Therapy for Rosacea. Front. Immunol..

[12] Rein A.S., Henke M., Brünner S., Luckhardt S., Zodel A.-L., Sethmann A., Schiffmann S. (2025). Influence of Sodium Bituminosulfonate and Doxycycline on Signal Molecules Relevant for Rosacea Symptoms. Sci. Rep..

[13] Rainer B.M., Kang S., Chien A.L. (2017). Rosacea: Epidemiology, Pathogenesis, and Treatment. Derm.-Endocrinol..

[14] Clanner-Engelshofen B.M., Bernhard D., Dargatz S., Flaig M.J., Gieler U., Kinberger M., Klövekorn W., Kuna A.-C., Läuchli S., Lehmann P. (2022). S2k Guideline: Rosacea. JDDG J. Dtsch. Dermatol. Ges..

[15] Schaller M., Almeida L.M.C., Bewley A., Cribier B., Rosso J.D., Dlova N.C., Gallo R.L., Granstein R.D., Kautz G., Mannis M.J. (2019). Recommendations for Rosacea Diagnosis, Classification and Management: Update from the Global ROSacea COnsensus 2019 Panel. Br. J. Dermatol..

[16] Sharma A., Kroumpouzos G., Kassir M., Galadari H., Goren A., Grabbe S., Goldust M. (2022). Rosacea Management: A Comprehensive Review. J. Cosmet. Dermatol..

[17] King S., Campbell J., Rowe R., Daly M.-L., Moncrieff G., Maybury C. (2023). A Systematic Review to Evaluate the Efficacy of Azelaic Acid in the Management of Acne, Rosacea, Melasma and Skin Aging. J. Cosmet. Dermatol..

[18] Feng X., Shang J., Gu Z., Gong J., Chen Y., Liu Y. (2024). Azelaic Acid: Mechanisms of Action and Clinical Applications. Clin. Cosmet. Investig. Dermatol..

[19] Sauer N., Oślizło M., Brzostek M., Wolska J., Lubaszka K., Karłowicz-Bodalska K. (2023). The Multiple Uses of Azelaic Acid in Dermatology: Mechanism of Action, Preparations, and Potential Therapeutic Applications. Adv. Dermatol. Allergol..

[20] Miyachi Y., Yamasaki K., Fujita T., Fujii C. (2021). Metronidazole Gel (0.75%) in Japanese Patients with Rosacea: A Randomized, Vehicle-controlled, Phase 3 Study. J. Dermatol..

[21] Jackson J.M., Knuckles M., Minni J.P., Johnson S.M., Belasco K.T. (2015). The Role of Brimonidine Tartrate Gel in the Treatment of Rosacea. Clin. Cosmet. Investig. Dermatol..

[22] Anderson M.S., Nadkarni A., Cardwell L.A., Alinia H., Feldman S.R. (2017). Spotlight on Brimonidine Topical Gel 0.33% for Facial Erythema of Rosacea: Safety, Efficacy, and Patient Acceptability. Patient Prefer. Adherence.

[23] Dall’Oglio F., Nasca M.R., Guglielmi G., Micali G. (2024). Scalp Rosacea Treated with Topical Ivermectin. Ski. Appendage Disord..

[24] Paichitrojjana A., Khuancharee K., Paichitrojjana A. (2025). Efficacy of Topical Ivermectin in Controlling Human *Demodex* Infestation: Evidence from Systematic Review and Meta-Analysis. Parasite Epidemiol. Control.

[25] Chen Y.J., Tang J., Wang L., Hua W. (2025). Sulfur and Its Derivatives in Dermatology: Insights Into Therapeutic Applications—A Narrative Review. J. Cosmet. Dermatol..

[26] van Zuuren E.J., Fedorowicz Z., Tan J., van der Linden M.M.D., Arents B.W.M., Carter B., Charland L. (2019). Interventions for Rosacea Based on the Phenotype Approach: An Updated Systematic Review Including GRADE Assessments. Br. J. Dermatol..

[27] Choe J., Barbieri J.S. (2023). Emerging Medical Therapies in Rosacea: A Narrative Review. Dermatol. Ther..

[28] Paichitrojjana A., Paichitrojjana A. (2023). Oral Isotretinoin and Its Uses in Dermatology: A Review. Drug Des. Dev. Ther..

[29] Assiri A., Hobani A.H., AlKaabi H.A., Mojiri M.E., Daghriri S.A., Suwaid O.A., Alameer M.I., Akkam M.M., Alamir M.A., Albarr A.A. (2024). Efficacy of Low-Dose Isotretinoin in the Treatment of Rosacea: A Systematic Review and Meta-Analysis. Cureus.

[30] Husein-ElAhmed H., Steinhoff M. (2021). Evaluation of the Efficacy of Subantimicrobial Dose Doxycycline in Rosacea: A Systematic Review of Clinical Trials and Meta-Analysis. JDDG J. Dtsch. Dermatol. Ges..

[31] Tao R.E., Prajapati S., Pixley J.N., Grada A., Feldman S.R. (2023). Oral Tetracycline-Class Drugs in Dermatology: Impact of Food Intake on Absorption and Efficacy. Antibiotics.

[32] Doan S., Gabison E., Chiambaretta F., Touati M., Cochereau I. (2013). Efficacy of Azithromycin 1.5% Eye Drops in Childhood Ocular Rosacea with Phlyctenular Blepharokeratoconjunctivitis. J. Ophthalmic Inflamm. Infect..

[33] Rodriguez-Cerdeira C., Sanchez-Blanco E., Molares-Vila A. (2012). Clinical Application of Development of Nonantibiotic Macrolides That Correct Inflammation-Driven Immune Dysfunction in Inflammatory Skin Diseases. Mediat. Inflamm..

[34] Alzolibani A.A., Zedan K. (2012). Macrolides in Chronic Inflammatory Skin Disorders. Mediat. Inflamm..

[35] Algarin Y.A., Pulumati A., Jaalouk D., Tan J., Nouri K. (2024). The Role of Vitamins and Nutrients in Rosacea. Arch. Dermatol. Res..

[36] Arman A., Demirseren D.D., Takmaz T. (2015). Treatment of Ocular Rosacea: Comparative Study of Topical Cyclosporine and Oral Doxycycline. Int. J. Ophthalmol..

[37] Callender V.D., Baldwin H., Cook-Bolden F.E., Alexis A.F., Stein Gold L., Guenin E. (2022). Effects of Topical Retinoids on Acne and Post-Inflammatory Hyperpigmentation in Patients with Skin of Color: A Clinical Review and Implications for Practice. Am. J. Clin. Dermatol..

[38] Sticchi A., Fiorito F., Kaleci S., Paganelli A., Manfredini M., Longo C. (2025). Rosacea and Treatment with Retinoids: A Systematic Review and Meta-Analysis. Ther. Adv. Chronic Dis..

[39] Sidiropoulou P., Katsarou M., Sifaki M., Papasavva M., Drakoulis N. (2024). Topical Calcineurin and Mammalian Target of Rapamycin Inhibitors in Inflammatory Dermatoses: Current Challenges and Nanotechnology-Based Prospects (Review). Int. J. Mol. Med..

[40] Gutfreund K., Bienias W., Szewczyk A., Kaszuba A. (2013). Topical Calcineurin Inhibitors in Dermatology. Part I: Properties, Method and Effectiveness of Drug Use. Adv. Dermatol. Allergol..

[41] Scala J., Vojvodic A., Vojvodic P., Vlaskovic-Jovicevic T., Peric-Hajzler Z., Matovic D., Dimitrijevic S., Vojvodic J., Sijan G., Stepic N. (2019). Botulin Toxin Use in Scars/Keloids Treatment. Open Access Maced. J. Med. Sci..

[42] Scala J., Vojvodic A., Vojvodic P., Vlaskovic-Jovicevic T., Peric-Hajzler Z., Matovic D., Dimitrijevic S., Vojvodic J., Sijan G., Stepic N. (2019). Botulin Toxin Use in Rosacea and Facial Flushing Treatment. Open Access Maced. J. Med. Sci..

[43] Gabbianelli R., Falcioni M.L., Nasuti C., Cantalamessa F., Imada I., Inoue M. (2009). Effect of Permethrin Insecticide on Rat Polymorphonuclear Neutrophils. Chem. Biol. Interact..

[44] Forton F.M.N. (2020). The Pathogenic Role of *Demodex* Mites in Rosacea: A Potential Therapeutic Target Already in Erythematotelangiectatic Rosacea?. Dermatol. Ther..

[45] Alenezi N., AlQusaimi R., Alajmi H., AlMutairi E.Y., Alenezi A.T., Saad A.R., Al Radhwan N. (2025). Permethrin Versus Benzyl Benzoate for the Treatment of Scabies: A Systematic Review and Meta-Analysis of Randomized Controlled Trials. Cureus.

[46] Abokwidir M., Feldman S.R. (2016). Rosacea Management. Ski. Appendage Disord..

[47] Logger J.G.M., Olydam J.I., Driessen R.J.B. (2020). Use of Beta-Blockers for Rosacea-Associated Facial Erythema and Flushing: A Systematic Review and Update on Proposed Mode of Action. J. Am. Acad. Dermatol..

[48] Page M.J., McKenzie J.E., Bossuyt P.M., Boutron I., Hoffmann T.C., Mulrow C.D., Shamseer L., Tetzlaff J.M., Akl E.A., Brennan S.E. (2021). The PRISMA 2020 Statement: An Updated Guideline for Reporting Systematic Reviews. BMJ.

[49] Mckenzie J., Brennan S., Ryan R., Thomson H., Johnston R., Thomas J. (2019). Chapter 3: Defining the Criteria for Including Studies and How They Will Be Grouped for the Synthesis. Cochrane Handbook for Systematic Reviews of Interventions.

[50] Sajdeh F., Samadi A., Naeimifar A., Yazdanparast T., Ahmadi M., Amiri F., Kassir M., Firooz A., Nasrollahi S.A. (2025). Efficacy and Safety of Oxymetazoline 1% Cream for the Treatment of Mild to Moderate Facial Rosacea. J. Cosmet. Dermatol..

[51] Sun Y., Man X., Xuan X., Huang C., Shen Y., Lao L. (2022). Tofacitinib for the Treatment of Erythematotelangiectatic and Papulopustular Rosacea: A Retrospective Case Series. Dermatol. Ther..

[52] Suggs A.K., Macri A., Richmond H., Munavalli G., Friedman P.M. (2020). Treatment of Erythematotelangiectatic Rosacea with Pulsed-Dye Laser and Oxymetazoline 1.0% Cream: A Retrospective Study. Lasers Surg. Med..

[53] Mendieta Eckert M., Landa Gundin N. (2016). Treatment of Rosacea with Topical Ivermectin Cream: A Series of 34 Cases. Dermatol. Online J..

[54] Ghoz M.T., Mohamed D.A., Ibrahim Z.A., Hassan G.F.R. (2021). Evaluation of the Efficacy and Safety of Platelet Rich Plasma Injection in Treatment of Rosacea. Dermatol. Ther..

[55] Huang H.-P., Hsu C.-K., Lee J.Y.-Y. (2021). Rosacea with Persistent Facial Erythema and High *Demodex* Density Effectively Treated with Topical Ivermectin Alone or Combined with Oral Carvedilol. Dermatol. Ther..

[56] Cameli N., Mariano M., Zanniello R., Berardesca E. (2020). Clinical and Noninvasive Instrumental Evaluation of the Efficacy of a Nonsteroidal Anti-Inflammatory 8-Beta Glycyrrhetinic Acid Cream for the Treatment of Erythema in Rosacea. Dermatol. Ther..

[57] Bageorgou F., Vasalou V., Tzanetakou V., Kontochristopoulos G. (2019). The New Therapeutic Choice of Tranexamic Acid Solution in Treatment of Erythematotelangiectatic Rosacea. J. Cosmet. Dermatol..

[58] Sharara M.A., Abdel Hamid K.S., Imam A.A. (2024). Efficacy of Single-Dose Oral Ivermectin in Treatment of Rosacea in Relation to Demodex Mites. Egypt. J. Dermatol. Venerol..

[59] Nobeyama Y., Aihara Y., Asahina A. (2023). Real-World Evidence for the Treatment of Rosacea with Sulfur or Metronidazole Preparation in Japanese Patients. JMA J..

[60] Aghaei M., Aghaei S., Behshadnia F., Ghomashlooyan M., Khaghani A., Baradaran E.H., Naeini F.F., Iraji F., Shahmoradi Z., Hosseini S.M. (2023). Association between the Treatment of Rosacea and Eradication of Helicobacter Pylori Infection. Adv. Biomed. Res..

[61] Dall’Oglio F., Lacarrubba F., Micali G. (2021). Erythema-Directed Digital Photography and Colorimeter Scores Correlate with Rosacea Erythema Evaluation in Patients under Treatment with Topical Ivermectin. Dermatol. Ther..

[62] Dall’Oglio F., Tedeschi A., Lacarrubba F., Fabbrocini G., Skroza N., Chiodini P., Micali G. (2021). A Novel Azelaic Acid Formulation for the Topical Treatment of Inflammatory Rosacea: A Multicentre, Prospective Clinical Trial. J. Cosmet. Dermatol..

[63] Hasanbeyzade S. (2025). Comparison of Topical 20% Azelaic Acid and 7.5% Dapsone in the Treatment of Mild-To-Moderate Papulopustular Rosacea. J. Cosmet. Dermatol..

[64] Gökşin Ş., İmren I.G., Kaçar N. (2024). Efficacy of Topical Dapsone 5% Gel for the Treatment of Erythematotelangiectatic Rosacea: New Treatment Option with Old Drug. Dermatol. Pract. Concept..

